# Multi-functional Small Molecule for Regenerative Healing of Avascular Meniscus Tears: Modulation of Inflammation, Differentiation, and Multi-Tissue Crosstalk

**DOI:** 10.7150/thno.132326

**Published:** 2026-04-08

**Authors:** Meng Feng, David D. Pellei, Ming Wang, Angelyn Nguyen, Sarah Ginsberg, Hun Jin Jeong, Chang H. Lee

**Affiliations:** College of Dental Medicine, Columbia University Irving Medical Center, 630 W. 168 St. - VC12-212, New York, NY 10032, USA.

**Keywords:** 4-PPBP, sigma-1 receptor, avascular meniscus healing, scRNA-seq, multi-tissue crosstalk

## Abstract

Avascular meniscus tears are a major contributor to mechanical joint locking, compromised gait and function, and the initiation and progression of post-traumatic osteoarthritis. Unfortunately, the avascular meniscus tears hardly heal. Here, we report a novel small molecule, 4-PPBP, a sigma-1 receptor (σ1R) agonist, exhibiting significant potential to promote avascular meniscus healing by activating synovial mesenchymal stem cells (syMSCs) and modulating macrophage-regulated inflammation via multi-tissue crosstalk. *In vitro,* 4-PPBP promoted the proliferation and migration of meniscus cells and syMSCs, exhibited anti-inflammatory effects, and induced fibrochondrogenic differentiation. 4-PPBP significantly promoted the healing of avascular meniscus tears *ex vivo*. *In vivo*, a single injection of 4-PPBP-loaded bioglue minimized meniscal gapping, with improved meniscus healing and gait performance. In contrast to the degenerative changes in the untreated control, 4-PPBP/bioglue application resulted in integrated fibrocartilaginous tissues. scRNA-seq and CellChat analyses revealed 4-PPBP-activated cell-cell communications leading to inflammatory regulation and cell differentiation. Macrophages showed a robust reduction in pro-inflammatory genes, and fibroblasts, chondrocytes, and fibrochondrocytes increased genes associated with differentiation and matrix synthesis in response to 4-PPBP. Anti-inflammatory cell-cell communication signals were significantly elevated between adipocytes and macrophages. Together, this study demonstrates the notable potential of 4-PPBP as a multi-functional therapeutic for avascular meniscus tears.

## Introduction

The knee menisci are fibrocartilaginous tissues in the joint that play essential roles in shock absorption, maintaining joint congruency and stability, and the distribution of synovial fluids 1, 2. Clinically, symptomatic meniscus tears are associated with reduced muscle mass and strength, limited daily activity, and altered walking patterns. Meniscus tears at the avascular zone require early surgical intervention, either meniscectomy or repair of the existing meniscus. However, tears at the avascular zone of the meniscus can be hardly repaired, leading to deterioration, followed by initiation and progression of osteoarthritis (OA) 3, 4. Meniscectomy also significantly increases the risk of OA by dramatically elevating cartilage contact pressure 5.

Biological augmentations have been explored to enhance clinical outcomes after meniscus tear repair. Fibrin clot, bone marrow aspirate concentrate, and platelet-rich plasma have been practiced in human patients, but their outcomes suffer from high variability and inconsistent long-term efficacy 6-10. Injectable hydrogels loaded with growth factors and various types of joint cells and stem/progenitor cells have been tested in animal models 11-16. Despite promising outcomes in meniscus healing, hydrogel injections with growth factors and cells face substantial translational barriers associated with the development cost and regulatory obstacles 17. Poor mechanical properties and quick intrasynovial degradation are among other limitations of the previous hydrogel systems 13, 18. Recently, we reported fibrin gel cross-linked with genipin (FibGen) as an injectable, mechanically stable, and slowly degrading hydrogel for delivering bioactive cues, leading to healing of avascular meniscus tears 13. We observed that FibGen sequentially releasing connective tissue growth factor (CTGF) and transforming growth factor beta 3 (TGF-β3) can promote functional restoration of meniscus tears by recruiting endogenous synovial mesenchymal stem/progenitor cells (syMSCs) and their differentiation 13-15, 19. However, our previous approach with multiple growth factors and controlled delivery vehicles is not free of the imminent translational barriers 20.

Besides biologics aiming to promote healing through metabolic activities, anti-inflammatory modalities have been investigated to mitigate the risk of degeneration and delayed healing 21-24. Upon meniscus injuries, synovial lining macrophages can stimulate synovial inflammation by releasing uncontrolled proinflammatory factors (e.g., IL-1β, TNFα, chemokines, and MMPs) 25. To address the pro-inflammatory signaling cascades mediated by synovial macrophages, anti-inflammatory biologics such as Leukocyte-Poor Platelet-Rich Plasma (LP-PRP), MSCs, and interleukin 1 receptor antagonist (IL-1Ra) have been investigated 26. Application of LP-PRP and MSCs reduced pain and inflammation after meniscus injury, but the long-term outcome was mixed 10. IL-1Ra has shown promising efficacy in reducing joint inflammation in pre-clinical models, but it failed to show a statistically significant improvement in human clinical trials 27, 28. These findings suggest that an anti-inflammation approach is necessary for slowing degenerative changes, but it is not sufficient to promote healing.

**Figure 1:**
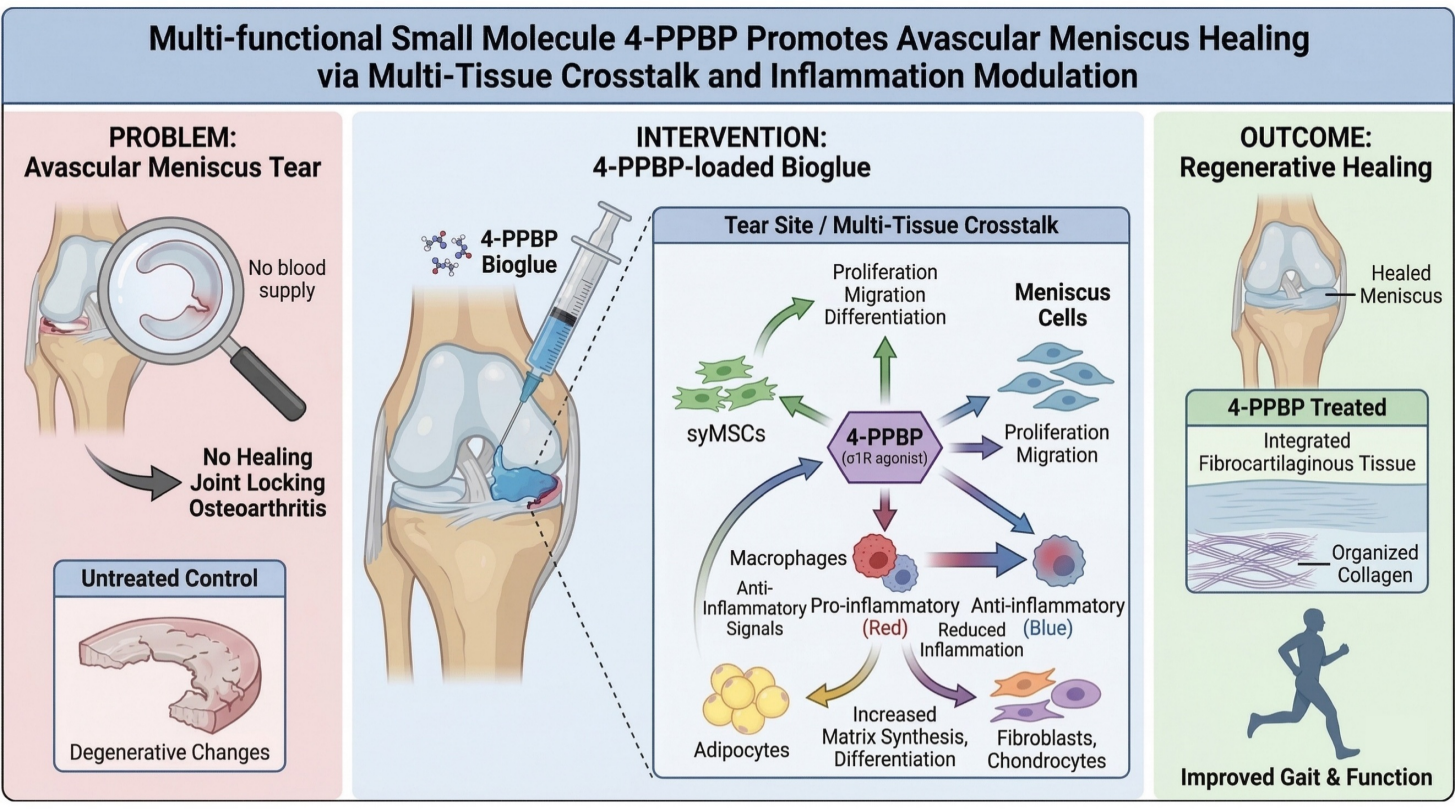
A multi-functional small molecule delivered via injectable bioglue to promote regenerative healing of meniscus tears.

In this study, we discovered a novel small molecule, 4-PPBP, with multiple functions of promoting avascular meniscus healing and mitigating inflammation (**Fig. 1**). As an ERK1/2 agonist, 4-PPBP has been shown to facilitate fibrogenic differentiation of MSCs 29, 30. In addition, 4-PPBP is an agonist of the sigma-1 receptor (σ1R) 30 and this study identified its anti-inflammatory functions through σ1R pathway. As σ1R is expressed in multiple cell types in the joint, including macrophages, immune cells, adipocytes, chondrocytes, and meniscus fibrochondrocytes 29, 31, 32, we explored the roles of 4-PPBP in anti-inflammation and meniscus healing through cell-cell crosstalk among these cell types. Our comprehensive scRNA-seq analysis with CellChat identified key signaling pathways involved in cell-cell communication that regulate macrophage polarization. Our findings demonstrate the notable potential of the multifunctional 4-PPBP, which guides regenerative healing of the meniscus by controlling inflammation and differentiation through multi-tissue crosstalk. As a single small molecule that can be controlled-delivered via a well-established injectable hydrogel, 4-PPBP may represent a novel regenerative therapeutic for avascular meniscus injuries.

## Results

### 4-PPBP promotes syMSC migration, proliferation, and fibrochondrogenic differentiation

**Figure 2:**
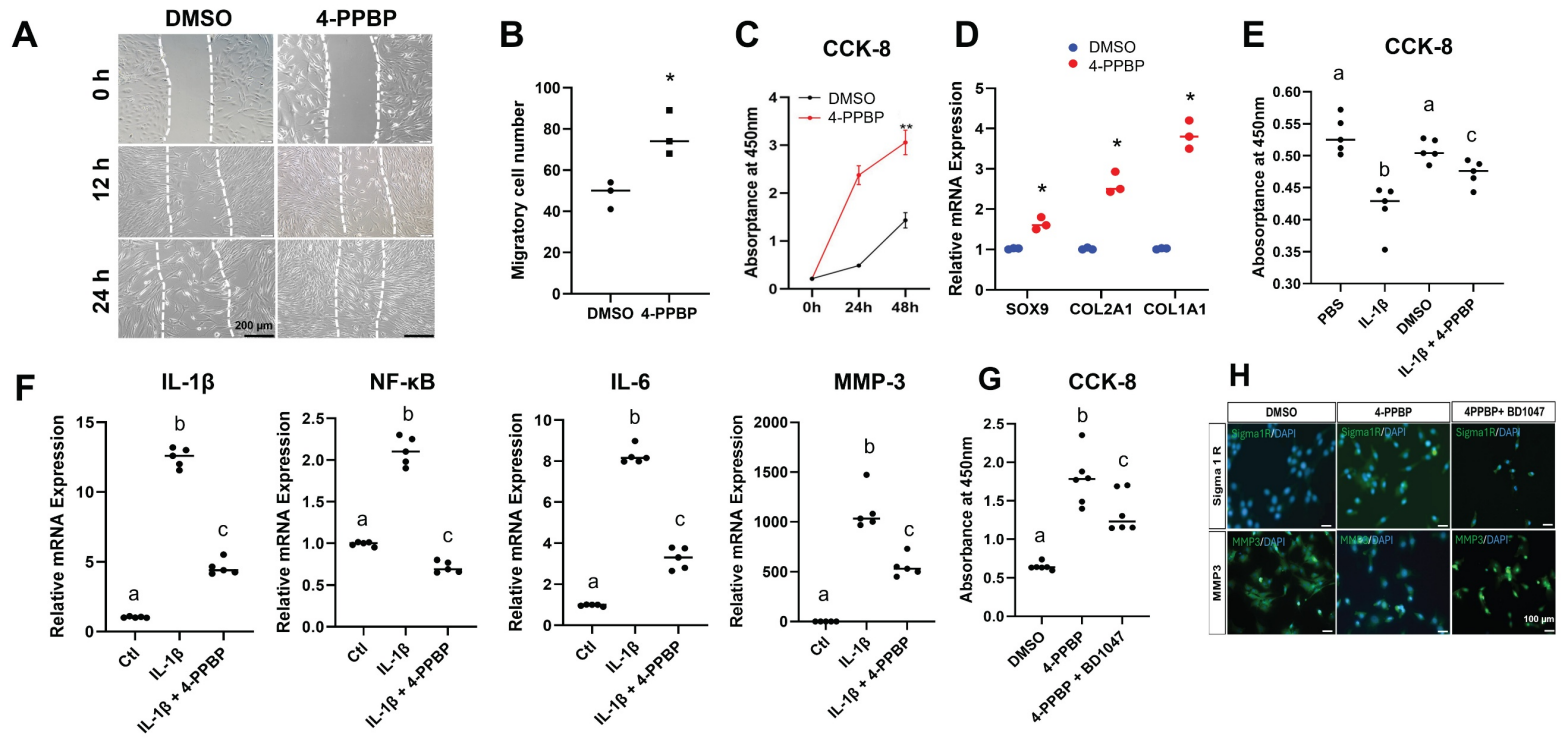
Multi-functional small molecule, 4-PPBP. In scratch wound healing assay, 4-PPBP promoted migration of syMSCs (**A, B**) (n=3 per group, *: p<0.01 compared to DMSO control). 4-PPBP also promoted proliferation of syMSC (**C**) (n=5 per group, **: p<0.001 compared to DMSO control). 4-PPBP enhanced fibrochondrogenic differentiation of syMSCs, with significantly elevated sox9, Col2a1, and Col1a1 expressions (**D**) (n=3 per group, *: p<0.001 compared to control). In addition, 4-PPBP significantly attenuated IL-1β-induced apoptosis in syMSCs at 24 hours (**E**). Similarly, pro-inflammatory gene expressions induced by IL-1β were significantly reduced by 4-PPBP treatment by 24 hours (**F**). Proliferation of syMSC by 4-PPBP was attenuated by BD1047 (**G**) (For **E-G**, n = 5 - 6 per group, p<0.001; different letters indicate statistically significant differences). Similarly, the expression of σ1R was elevated by 4-PPBP, which was reduced by BD1047 (**H**). In contrast, MMP-3 expression reduced by 4-PPBP was recovered by BD1047 (**H**), confirming the functions of 4-PPBP are likely mediated through σ1R.

As the primary endogenous cell sources for meniscus regeneration 7, we tested the effects of 4-PPBP on syMSCs and meniscus fibrochondrocytes *in vitro*. The scratch wound healing model showed that 4-PPBP (10 µM in DMSO) promoted migration of human syMSCs (**Fig. 2A & B**) as compared to DMSO control. The CCK-8 assay suggested that 4-PPBP stimulates syMSC proliferation for 48 hours (**Fig. 2C**). The treatment of 4-PPBP also induced fibrochondrogenic differentiation of syMSCs, with significantly increased Sox9, Col2A1, and Col1A1 expressions by 48 hours as compared to control (**Fig. 2D**).

### Anti-inflammatory function of 4-PPBP

Treatment with 4-PPBP significantly attenuated IL-1β-induced apoptosis in syMSCs at 24 hours (**Fig. 2E**). Expression of pro-inflammatory cytokines and enzymes, including IL-1β, NF-κB, IL-6, and MMP-3, was significantly increased by IL-1β treatment, but was significantly reduced with 4-PPBP treatment (**Fig. 2F**).

### σ1R inhibition interrupts the effects of 4-PPBP

To confirm the 4-PPBP function through σ1R signaling, a small molecular σ1R inhibitor, BD1047 (200 µM), was applied. BD1047 significantly reduced the proliferation of syMSCs induced by 4-PPBP (**Fig. 2G**). Immunofluorescence showed enhanced expressions of σ1R on syMSCs by 4-PPBP treatment for 24 hours, which was reduced by BD1047 (**Fig. 2H**). Similarly, MMP-3 expressions attenuated by 4-PPBP were reversed by BD1047 (**Fig. 2H**). These findings suggest that multi-functions of 4-PPBP are regulated by σ1R signaling.

### Effect of 4-PPBP on human and bovine meniscus cells

The treatment of 4-PPBP to human and bovine meniscus cells significantly increased the proliferation by 24 hours (**Supplementary Fig. 1A, C**). BD1047 attenuated the effect of 4-PPBP, and IL-1β-reduced cell viability was rescued by 4-PPBP (**Supplementary Fig. 1A, C**). In human meniscus cells, Col2a1 and Col1a1 were significantly reduced by IL-1β (**Supplementary Fig. 1B**). Col2a1 expression was significantly increased by 4-PPBP treatment (**Supplementary Fig. 1B**). In bovine meniscus cells, all the tested fibrochondrogenic genes, including Sox9, Col2a1, and Col1a1, were significantly reduced by IL-1β in bovine meniscus cells, and Sox9 and Col1a1 were rescued by 4-PPBP (**Supplementary Fig. 1D**).

### 4-PPBP promotes healing of avascular meniscus injuries in an explant model

**Figure 3:**
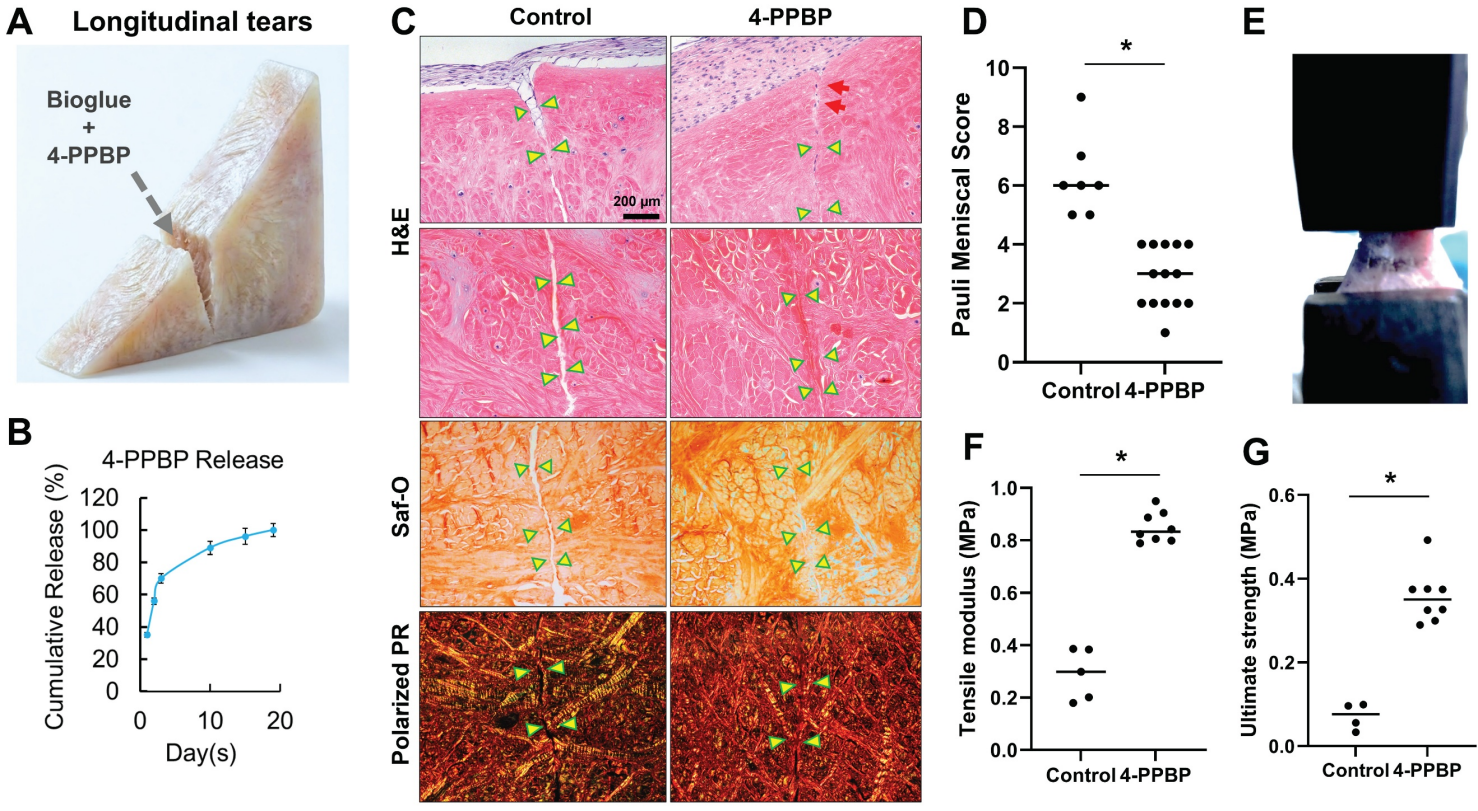
4-PPBP promotes healing of avascular meniscus injuries *ex vivo*. Wedge-shaped explants of inner menisci were prepared from bovine, followed by applying FibGen with 10 µM of 4-PPBP (**A**). 4-PPBP showed a sustained release from FibGen bioglue up to 18 days *in vitro* (**B**). Consistently, Pauli scores were significantly lower with 4-PPBP treatment (**D**) (n = 6 - 14 per group; *: p<0.001). Tensile tests performed with the healed meniscus explants (**E**) resulted in significantly higher tensile modulus (**F**) and ultimate strength (**G**) with 4-PPBP treatment as compared to the control (n=4-8 per group, *: p<0.001).

The efficacy of 4-PPBP in healing avascular meniscus tears was tested in our well-established meniscus explant healing model 13-15. Full thickness of longitudinal or radial incision was created in the inner third zone of bovine meniscus explants. Then, fibrin cross-linked with genipin (FibGen: 100 mg/ml fibrinogen + 100 U/ml thrombin + 2.5 mg/ml genipin), loaded with 10 µM of 4-PPBP, was applied into the explant defect (**Fig. 3A**). 4-PPBP loaded in FibGen showed a sustained release up to 18 days (**Fig. 3B**). After applying 4-PPBP/FibGen, 1 M/ml syMSCs were plated surrounding the explants per our previous methods 13, 14. Application of 4-PPBP/FibGen recruited syMSCs into the defect (**Fig. 3C**), followed by enhancing integrated healing of meniscus defects by 4 wks as compared to control with FibGen alone and syMSCs (**Fig. 3C**). Quantitatively, Pauli meniscus scores were significantly lower with 4-PPBP/FibGen than the control with syMSCs and FibGen bioglue (**Fig. 3D**). Consistently, tensile tests performed with the healed meniscal explants (**Fig. 3E**) showed significantly higher tensile modulus and ultimate strength with 4-PPBP/FibGen as compared to the control (**Fig. 3F, G**). Radial tears treated by 4-PPBP/FibGen also showed promoted healing as compared to the control (**Supplementary Fig. 2**).

### *In vivo* efficacy of 4-PPBP in regenerative healing of avascular meniscus tears

We tested the *in vivo* efficacy of 4-PPBP using a validated longitudinal tear model in rats 14. After creating a longitudinal tear in the anterior horn of medial meniscus, 20 µl bioglue (FibGen) loaded with 10 µM of 4-PPBP was applied to the tear. Untreated control and bioglue alone were included with a group adding BD1047 (200 µM) (**Supplementary Fig. 3**).

**Figure 4:**
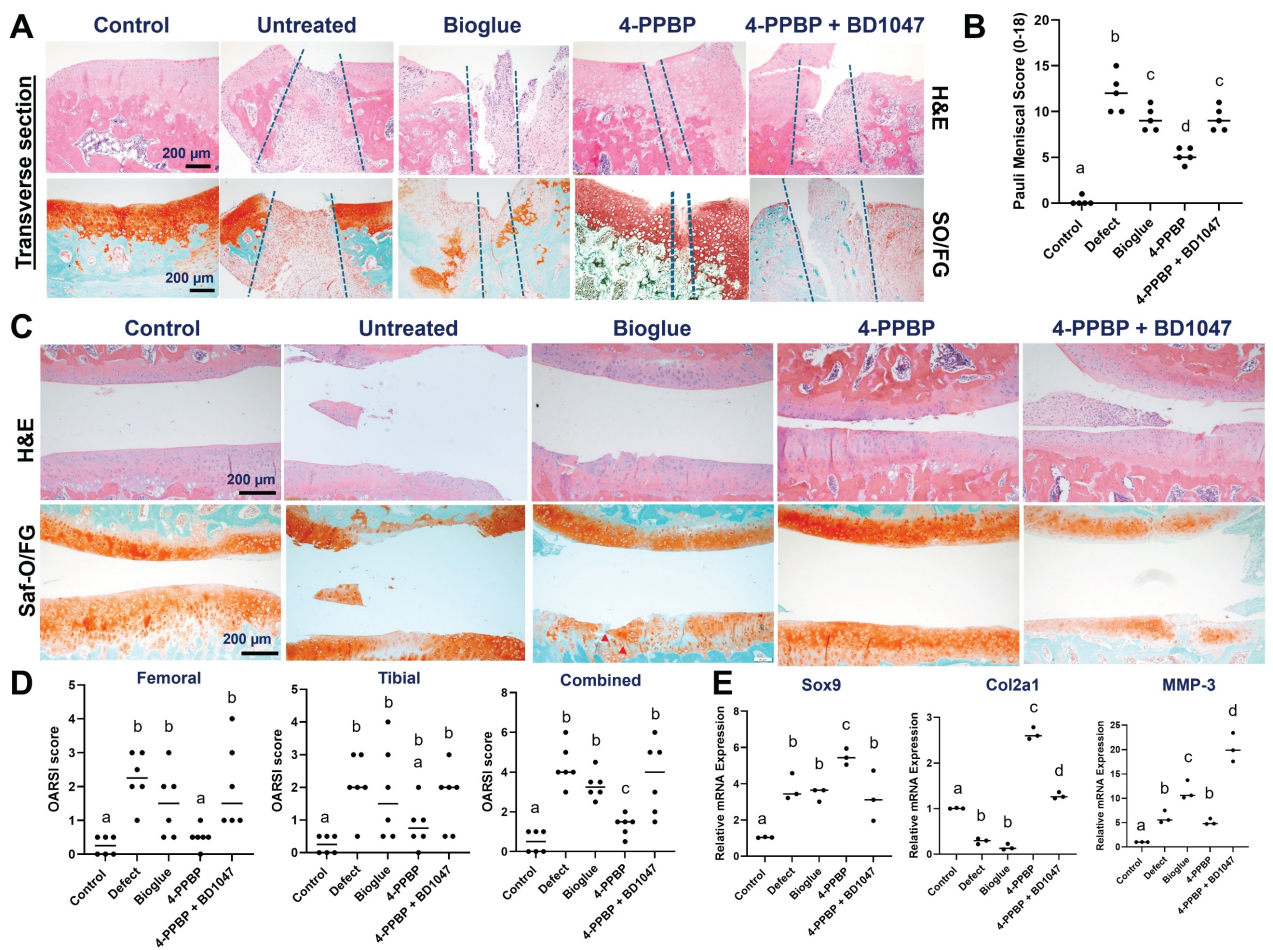
Rat meniscus healing by 4-PPBP treatment by 2 weeks post-op. Transverse tissue sections (**A**) revealed integrative healing of meniscus with 4-PPBP delivery, while all the other groups, untreated control, bioglue alone, and 4-PPBP + BD1047 showed poor healing with degenerative changes (**A**). Semi-quantitative Pauli scores were significantly lower with 4-PPBP (**B**) (n = 5 per group). Articular cartilages remained intact with 4-PPBP, comparable to sham control (**C**). However, untreated control and bioglue alone exhibited damage on cartilage (**C**). 4-PPBP + BD1047 showed signs of degradation of cartilaginous matrix (**C**). OARSI scores were significantly lower in 4-PPBP than all the other groups (**D**) (n=6 per group). qRT-PCR showed significantly increased sox9 and Col2a1 expressions with 4-PPBP, and reduced MMP-3 expression (**E**) (n = 3 per group). In B, D, and E, p<0.001; different letters indicate significant differences.

By 2 wks post-op, macroscopic observation revealed enhanced healing with 4-PPBP delivery compared to untreated control, bioglue alone, and BD1047 groups (**Supplementary Fig. 4**). Transverse sections of harvested menisci showed an integrated fibrocartilaginous healing with 4-PPBP treatment (**Fig. 4A**). However, untreated control, bioglue alone, and BD1047 treatment showed degenerative changes with scarred healing (**Fig. 4A**). Quantitatively, Pauli meniscus scores were significantly lower with 4-PPBP delivery than all the other surgical groups (**Fig. 4B**). Articular cartilage remained intact with 4-PPBP delivery, but untreated control, bioglue alone, and BD1047 groups showed damage on the cartilages (**Fig. 4C**). Consistently, OARSI scores were significantly lower with 4-PPBP delivery in comparison with the other surgical groups (**Fig. 4D**). qRT-PCR performed using all joint cells showed significant increases in Sox9 and Col2A1 and a significant reduction of MMP-3 with 4-PPBP (**Fig. 4E**).

**Figure 5:**
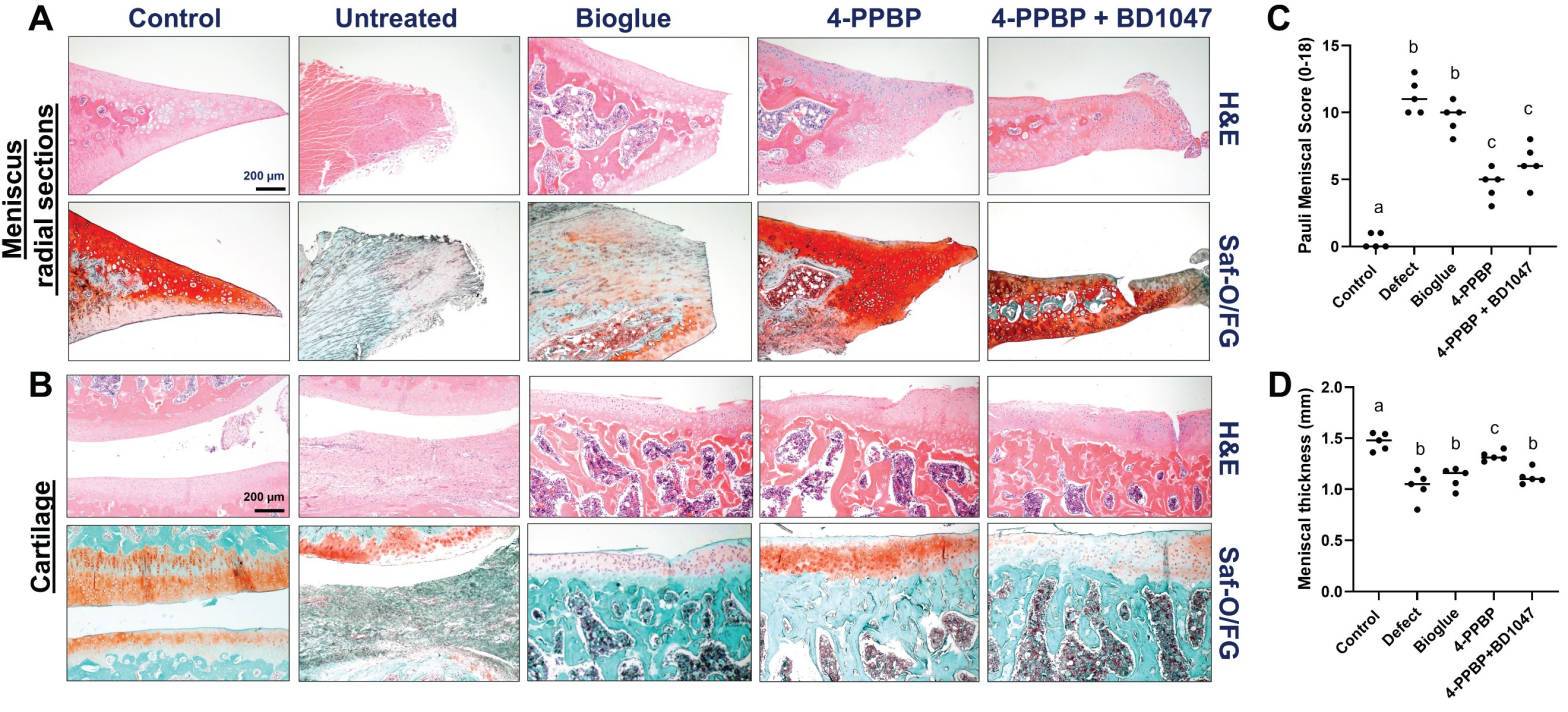
Rat meniscus healing with 4-PPBP treatment by 4 weeks post-op. Radial tissue sections of harvested menisci showed integrated healing with 4-PPBP in contrast to remaining gap with degenerative changes in the other groups (**A**). Cartilage histology (**B**) showed a remained tissue integrity with 4-PPBP, while all the other groups showed signs of degenerative changes (Saf-O/FG: safranin O and fast green). The Pauli meniscus score was significantly lower with 4-PPBP (**C**) (n = 5 per group). Meniscus thickness was significantly higher with 4-PPBP, closer to sham control (**D**) (n = 5 per group). p<0.001; different letters indicate significant differences.

By 4 weeks post-op, macroscopic evaluation suggests a tissue closure in 4-PPBP, while remaining gaps in untreated defect, bioglue, and 4-PPBP + BD1047 groups (**Supplementary Fig. 5**). Macroscopic images on joint tissues appear to exhibit tissue inflammation in the defect, bioglue, and 4-PPBP + BD1047 groups, in contrast to 4-PPBP (**Supplementary Fig. 5**). Histologically, radial sections of the harvested menisci showed integrated fibrocartilaginous healing of meniscus tears with 4-PPBP delivery (**Fig. 5A**). Untreated control and bioglue showed failed tissue integration and notable degenerative changes with loss of cartilaginous matrix (**Fig. 5A**). BD1047 showed poor tissue integration but maintained fibrocartilaginous matrix (**Fig. 5A**). 4-PPBP treatment also maintained the cartilage integrity, comparable to sham control (**Fig. 5B**). Bioglue alone and 4-PPBP + BD1047 showed loss of cartilaginous matrix, while untreated defect showed signs of degeneration of joint structure with scarred tissue formation (**Fig. 5B**). Quantitatively, Pauli meniscus scores were significantly lower with 4-PPBP delivery (**Fig. 5C**). In addition, meniscus thickness was significantly higher with 4-PPBP than other groups (**Fig. 5D**).

### Functional restoration

**Figure 6:**
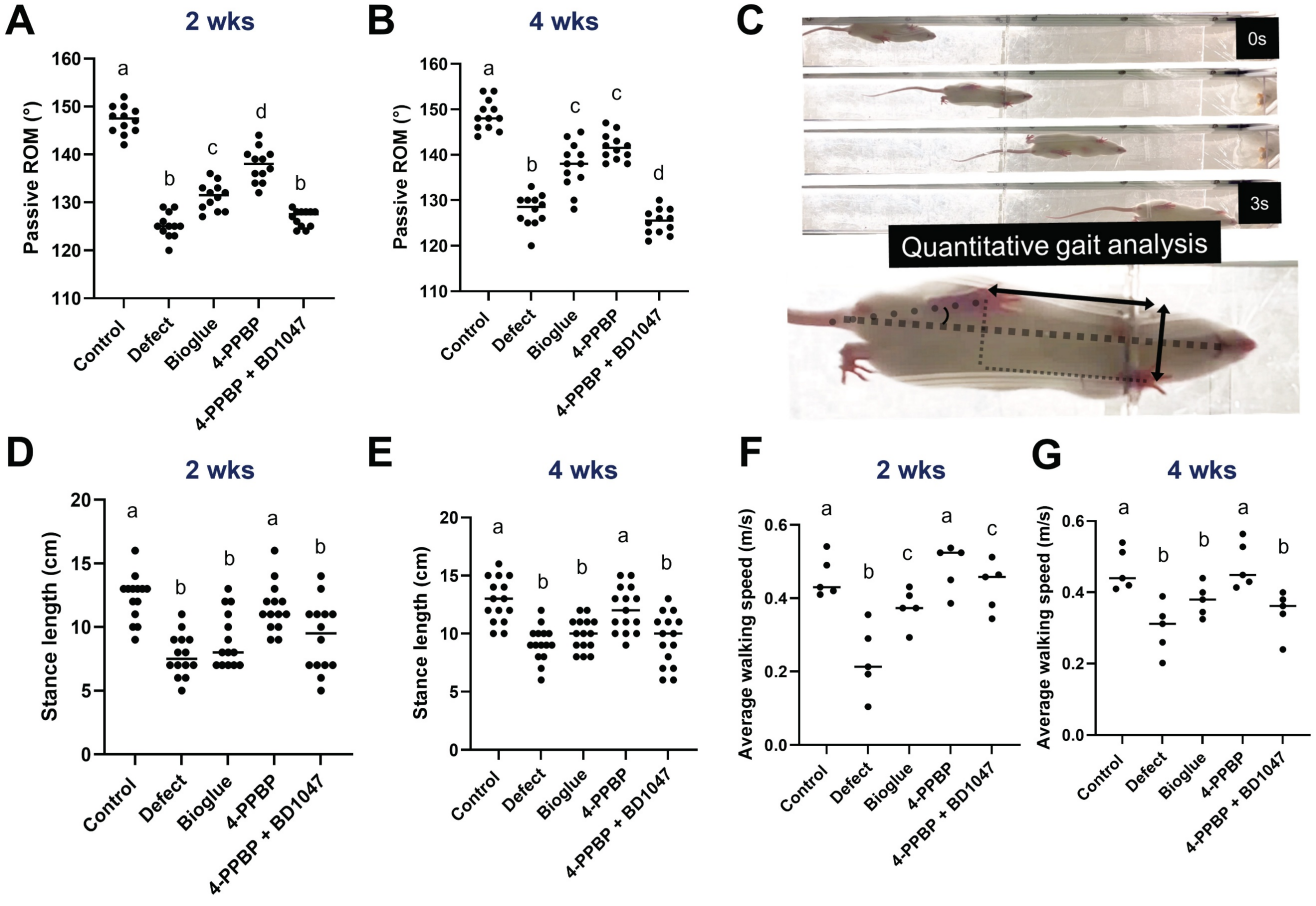
Functional evaluation by 4 weeks post-op. Passive ROM was significantly higher with 4-PPBP in 2 weeks (**A**). In 4 weeks, bioglue alone and 4-PPBP showed higher ROM than defect and 4-PPBP + BD1047 (**B**) (n = 11 - 12 per group). Gait analysis performed by a custom Catwalk system (**C**) resulted in stance length significantly reduced by meniscus defect by 2 and 4 weeks, which was significantly recovered by 4-PPBP treatment (**D, E**) (n = 11 - 13 per group). Average walking speed reduced by meniscus defect was significantly recovered by 4-PPBP delivery by 2 and 4 weeks post-op (**F, G**) (n = 5 per group). p<0.001; different letters indicate significant differences.

The recovery of joint functions was evaluated by passive range of motion (ROM) and gait analysis. Significantly larger ROM was observed with 4-PPBP by 2 weeks and 4 weeks post-op, as compared to the other surgical groups (**Fig. 6A & B**). Gait analysis performed using a custom-built CatWalk system (**Fig. 6C**). By 2- and 4-weeks post-op, the average stance length was significantly reduced by meniscus injury, which was recovered by 4-PPBP delivery (**Fig. 6D**). Similarly, the average walking speed was significantly higher with 4-PPBP delivery than all the other surgical groups by 2 and 4 weeks post-op (**Fig. 6E**).

### scRNA-seq and CellChat analysis reveal cell-cell communications regulated in 4-PPBP-induced meniscus healing

**Figure 7:**
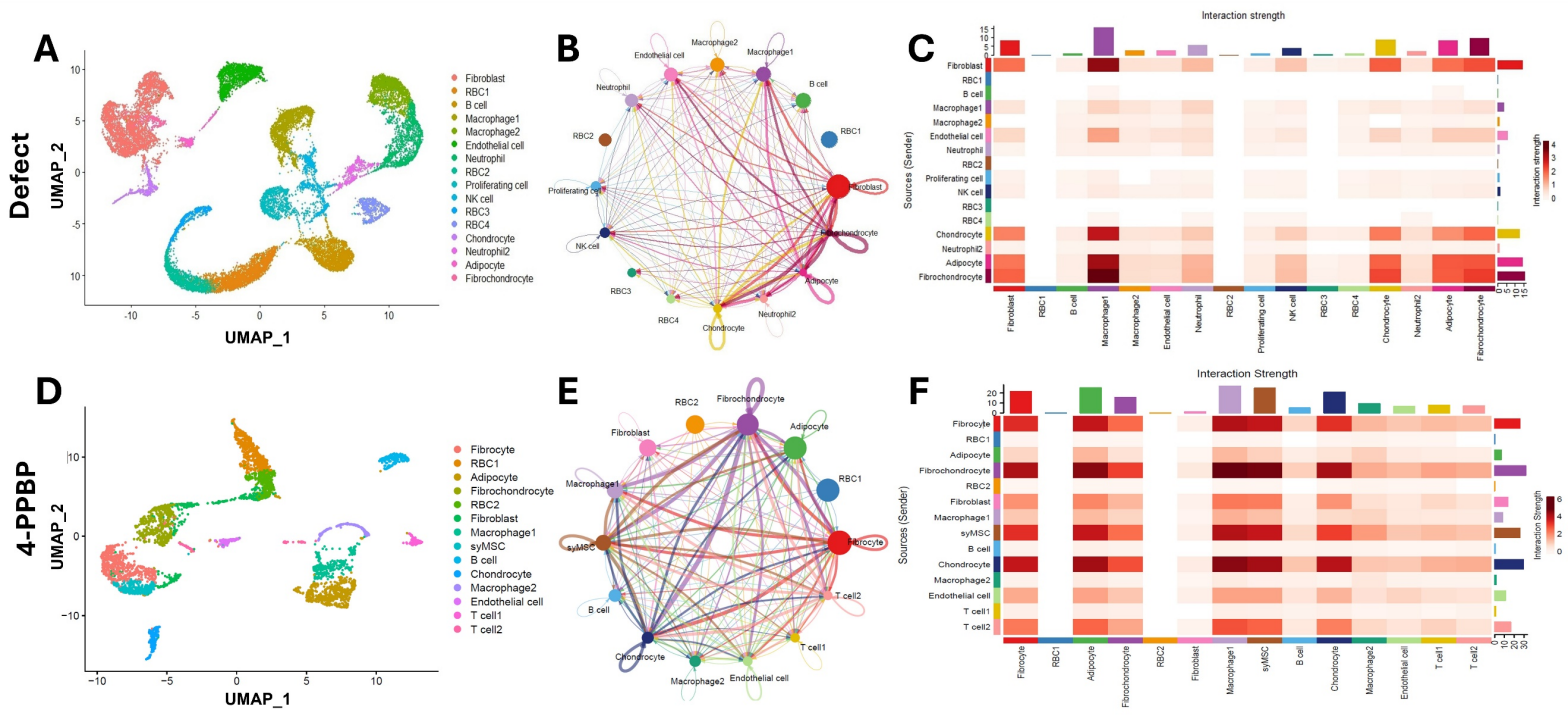
scRNA-seq analysis of knee joint cells at 2 weeks post-op. UMAPs show multiple cell clusters identified in the samples from untreated defect and 4-PPBP treatment groups (**A, D**). CellChat analysis resulted in circle plots displaying intensity of cell-cell communication interactions between different types of cells (**B, E**). Heatmaps for interaction strengths of outgoing and incoming signals confirm that fibroblast, macrophage, fibrochondrocyte, adipocyte, and chondrocyte are the primary cell types involved in strong cell-cell communication (**C, F**).

Cells isolated from knee joint tissues at 2 weeks post-op were used for scRNA-seq using 10X Genomics Next Generation Sequencing (NGS). UMAP showed multiple cell clusters identified in the joint tissues, including but not limited to fibrochondrocytes, chondrocytes, fibroblasts, macrophages, and immune cells (**Fig. 7A, D**). The cluster of syMSCs, exhibiting features of undifferentiated MSCs (e.g., CD90, CD146, and CD105) with traits of differentiation into fibrochondrocytes (e.g., prg4, col1a1, col2a1, and sox9), was only identified in the 4-PPBP-treated group, not the untreated control (**Fig. 7A & D**), likely suggesting the recruitment of syMSCs into the meniscus defect consistently with our *ex vivo* data. CellChat analysis described cell-cell communication among different cell types in joint tissues. Circle plots show intensity of the interactions between cell types (**Fig. 7B & E**). Heatmaps were produced to visualize the strengths of interactions between cell pairs (**Fig. 7C & F**). In both control and 4-PPBP groups, fibrochondrocytes, fibroblasts, chondrocytes, and macrophages showed robust communication with each other (**Fig. 7C & F**). In the 4-PPBP group, syMSCs showed strong interactions with fibrochondrocytes and chondrocytes (**Fig. 7F**).

To describe significantly expressed cell-cell communication signals to & from syMSCs, bubble plots were created (**Supplementary Fig. 7**). Collagen and fibronectin signals, associated with fibrochondrogenic differentiation 33, were significantly expressed from fibrochondrocytes, fibroblasts, and chondrocytes to syMSCs (**Supplementary Fig. 6A**). Interestingly, Pdgfa/c-Pdgfra axis was observed from fibrochondrocytes to syMSCs, suggesting the potential role of fibrochondrocytes in recruiting syMSCs into the defect area (**Supplementary Fig. 6A**). Interesting outcoming signals from syMSCs include Cxcl1-Cxcr2 and Cxcl3-Cxcr2 to macrophages, suggesting syMSCs may be involved in recruiting macrophages (**Suppl Fig. 6B**). These signaling pathways between syMSCs and other cells were not quantitatively compared with the untreated group, as the control failed to show sufficient number of syMSCs.

**Figure 8:**
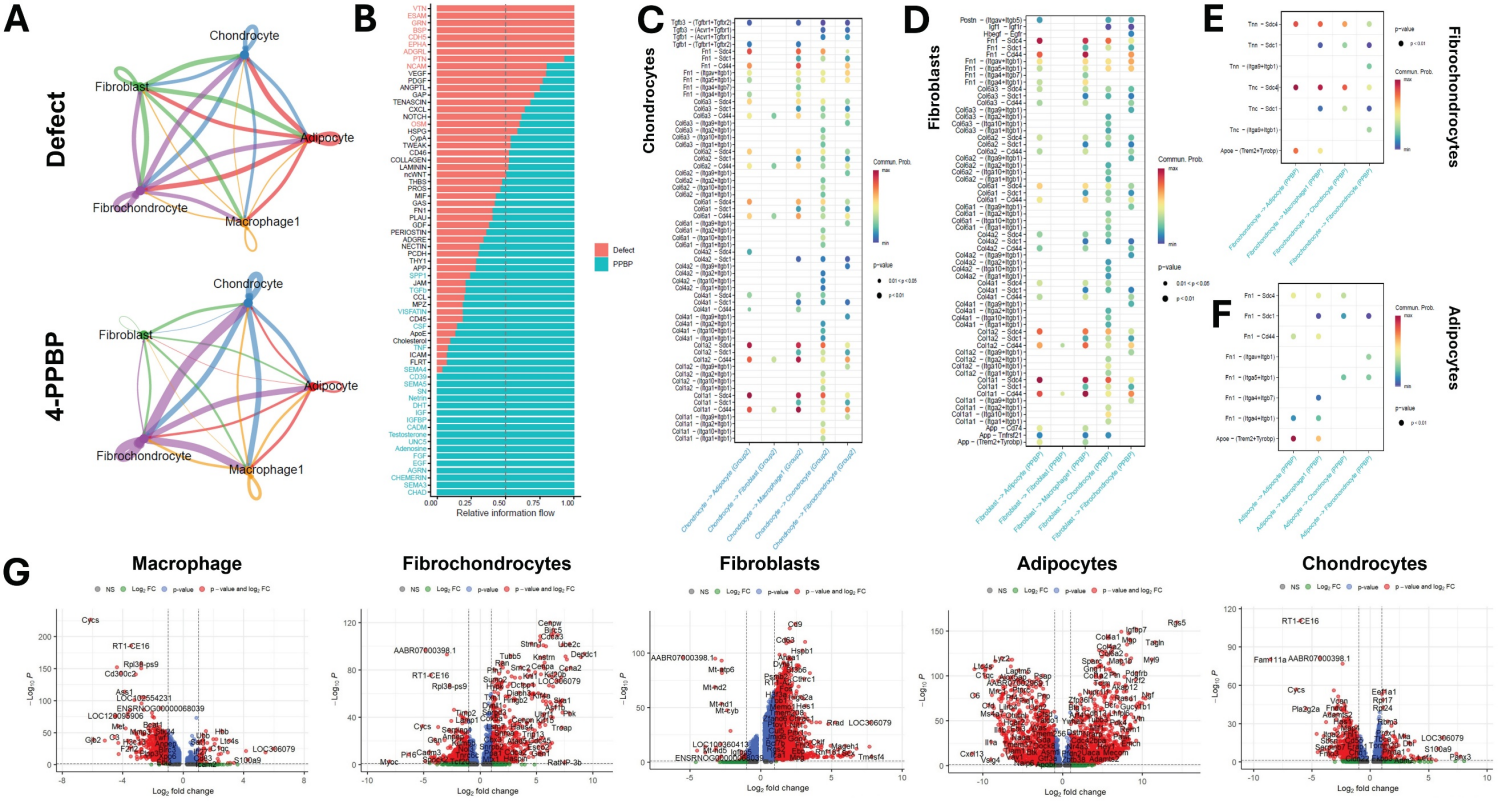
Comparative CellChat analysis was performed between the untreated control and 4-PPBP for a selected subset of cells, fibroblast, chondrocyte, adipocyte, macrophage, and fibrochondrocyte. Circle plots (**A**) exhibit notably strong interactions among chondrocytes, adipocytes, and fibrochondrocytes in the 4-PPPB compared to control. Quantitative analysis of the overall signal flow (**B**) revealed that, in 4-PPBP, signal pathways associated with inflammation and OA onset are reduced, and the signals associated with fibro/cartilaginous matrix synthesis, regeneration, protection against OA are increased. Quantitative comparison of ligand-receptor expressions between the defect and 4-PPBP showed that 4-PPBP significantly increased collagen and fibronectin signaling coming from chondrocytes and fibroblasts (**C & D**). 4-PPBP-upregulated cell-cell communication signals from fibrochondrocyte (**E**) include ApoE-(Trem2+Tyrobp), Tnc-Sdc4, and Tnn-Sdc4 axes to adipocytes, and Tnc-Sdc4 and Tnn-Sdc4 axes toward macrophages (**E**). 4-PPBP-upregulated cell-cell communication signals from adipocytes include ApoE to macrophages (**F**). Differentially expressed genes (DEG) analysis (**G**) confirmed significant reductions in pro-inflammatory genes in macrophages, fibrochondrocytes, and adipocytes with 4-PPBP treatment. OA-related genes were significantly reduced in chondrocytes with 4-PPBP (**G**).

Then we performed a comparative CellChat analysis between the untreated control and 4-PPBP for the five cell types involved in major cell-cell communication in both groups: fibrochondrocytes, fibroblasts, chondrocytes, and macrophages. Circle plots display distinct signaling strengths between fibrochondrocytes, chondrocytes, adipocytes, and macrophages (**Fig. 8A**). The 4-PPBP treatment group shows more intense interactions in fibrochondrocyte-chondrocyte, fibrochondrocyte-macrophage, and fibrochondrocyte-adipocyte as compared to the control (**Fig. 8A**). Quantitative comparison of each signaling flow revealed that signal pathways associated with inflammation and OA onset, such as vitronectin (VTN), cadherin-5 (CDH5), and pleiotrophin (PTN) (**Fig. 8B**). In contrast, 4-PPBP treatment showed high expressions of signaling associated with fibro/cartilaginous matrix synthesis, regeneration, protection against OA, such as chondroadherin (CHAD), agrin (AGRN), epidermal growth factor (EGF), fibroblast growth factor (FGF), insulin-like growth factor (IGF), and TGFβ (**Fig. 8B**). Lipid metabolic signaling, apolipoprotein E (ApoE), and a syMSC marker, THY1, were also relatively higher in the 4-PPBP group (**Fig. 8B**).

Quantitative comparison of ligand-receptor expressions revealed that 4-PPBP significantly increased collagen and fibronectin signaling coming from chondrocytes and fibroblasts compared with the control (**Fig. 8C & D**). Interestingly, 4-PPBP treatment significantly increased ApoE-(Trem2+Tyrobp), Tnc-Sdc4, and Tnn-Sdc4 axes from fibrochondrocytes to adipocytes (**Fig. 8E**), which are associated with lipid metabolism and protection against inflammation 34-36. Fibrochondrocytes with 4-PPBP treatment also showed significantly increased signals of the Tnc-Sdc4 and Tnn-Sdc4 axes toward macrophages (**Fig. 8E**), with reported functions in anti-inflammation and in promoting M2 polarization 37. ApoE signaling was also significantly increased in adipocytes-to-macrophages (**Fig. 8F**), which are reported to induce the anti-inflammatory M2 macrophage phenotype 38. These findings suggest that fibrochondrocytes and adipocytes play anti-inflammatory roles in the 4-PPBP-treated joint through crosstalk with macrophages. Consistently, differentially expressed genes (DEG) analysis confirmed significant reductions in pro-inflammatory genes in macrophages (e.g., Cd300c2, RT1-CE16, Ass1, and MMP-3) 39, 40 (**Fig. 8G**). Similarly, pro-inflammatory genes were significantly reduced in fibrochondrocytes and adipocytes with 4-PPBP treatment (**Fig. 8G**). Chondrocytes significantly reduced the expression of OA-related genes, such as Fndc1, Has1, and Pla2g2a 41, with 4-PPBP therapy as compared to the control (**Fig. 8G**).

### Effect of 4-PPBP on adipocytes *in vitro*

Given robust cell-cell communication from adipocytes to macrophages, likely activated by 4-PPBP treatment, we performed an *in vitro* experiment to confirm the direct or indirect effects of 4-PPBP on adipocytes. Adipose tissues engineered from adipose tissue derived mesenchymal stem/progenitor cells (ADSCs) for 3 weeks per our established methods 42, 43 were treated by 10 µM 4-PPBP. After 48 hours, qRT-PCR was performed for pro-inflammatory genes (IL-1β, NF-κB, and MMP-3) and adipogenic genes (ADIPOQ, LEPTIN, and PPARG). As result, pro-inflammatory gene expressions were significantly reduced in adipocytes by 4-PPBP treatment (**Supplementary Fig. 7A**). However, the expressions of adipogenic markers were not significantly affected by 4-PPBP treatment (**Supplementary Fig. 7B**). These observations suggest that 4-PPBP has a direct anti-inflammatory effect on adipocytes, but the adipogenic differentiation/metabolism observed *in vivo* may be derived from systemic interactions rather than a direct effect.

### *In vitro* validation of cell-cell communication signals

The effect of 4-PPBP on increased expressions of ApoE, Tn-C, and Tn-N in fibrochondrocytes was evaluated in vitro. Human fibrochondrocytes treated with 10 µM 4-PPBP for 24 hours significantly increased the expressions of ApoE and Tn-C (**Supplementary Fig. 8**), validating the CellChat inferred cell-cell communication signaling activated by 4-PPBP delivery *in vivo*. In contrast, Tn-N expression was not detected in fibrochondrocytes with or without 4-PPBP (**Supplementary Fig. 8**), suggesting an indirect, systemic route of Tn-N induction from 4-PPBP.

## Methods

### Materials

4-Phenyl-1-(4-phenylbutyl) piperidine maleate (4-PPBP) (#0620, Tocris Bioscience) was diluted to 50 mM in DMSO to make a stock solution. Then, 4-PPBP/DMSO was diluted to the working concentration (10 µM) with PBS. BD1047 (#0883, Tocris Bioscience), σ1R inhibitor, was prepared with PBS at a final concentration of 200 µM. FibGen bioglue was prepared with 100 mg/ml fibrinogen and 100 U/ml thrombin, cross-linked with 2.5 mg/ml genipin per our previous methods 13. All the other chemicals were purchased from Millipore Sigma (Burlington, MA, USA) unless otherwise stated. DMEM, MEM-a, fetal bovine serum, penicillin, streptomycin, trypsin, and PCR reagents and primers were purchased from Thermo Fisher Scientific (Waltham, MA, USA). The dose of 4-PPBP, 10 μM, was pre-optimized as an effective and safe dose from our pilot studies and previous works 30.

### Primary cell isolation and cell culture

Primary meniscus fibrochondrocytes were isolated from healthy bovine knee joints (#8-240128, Animal Technologies) and from human meniscus post-meniscectomy (NDRI; 64 years old, female), using type 2 collagenase for 6 hours, followed by growth of migratory cells for 24 hours. Human syMSCs (56-year-old female patient) (Articular Engineering, Northbrook, IL) were characterized and validated by flow cytometry, as described in our prior methods 33, 42-44. Adipose tissue derived stem/progenitor cells (ADSCs) purchased from Lonza (Morristown, NJ) were induced to differentiate into adipocytes for 4 weeks using adipogenic differentiation media per our prior methods 33, 42, 43.

### Proliferation and migration assay

To determine cell proliferation in the presence of IL-1β, 4-PPBP, or BD1047, Cell Counting Kit-8 (CCK-8) was used to assess cell viability. Briefly, P2-3 10,000 human syMSCs or bovine meniscus cells were seeded into 96-well plates and treated by IL-1β, 4-PPBP or 4-PPBP + BD1047, followed by incubation at 37 °C and 5% CO_2_ in a humidified incubator overnight. Then 10 μL of cell CCK-8 solution (#96992, Sigma-Aldrich, MO, USA) was added to each well. After incubation for 2 h, the absorbance at 450 nm was measured with a microplate reader. The migration of syMSCs were assayed using a scratch wound healing model following previously described method 45. Briefly, we used >90% confluent syMSCs (25x10^3^ cells per well) and then created a scratch wound, and the number of migrating cells filling up wound was counted.

### Anti-inflammatory effects of 4-PPBP

The anti-inflammatory effect of 4-PPBP was tested in human and bovine meniscus cells, and human syMSCs treated with 10 ng/ml IL-1β and/or 10 µM 4-PPBP. After 24 hours, the expression of inflammatory markers was measured by qRT-PCR.

### qRT-PCR

The total RNA was extracted using Qiagen RNAeasy mini kit (#74104, Qiagen, Germany) according to the manufacturer's instructions. RNA concentration and purity were determined by using a NanoDrop spectrophotometer. A 500 ng RNA was used to synthesize complementary DNA using a reverse transcription reagents kit (#433182, Thermo Fisher, Waltham, MA, USA). The real-time quantitative PCR was conducted with a ViiA7 real-time system (Thermo Fisher, Waltham, MA, USA) for markers, including IL-1β, IL-6, TNF-α, MMP-3, SOX9, COL1A1, COL2A1, ACAN, ADIPOQ, Leptin, NF-κB, and PPARG. The cycle threshold (Ct) values for GAPDH normalization and samples were measured by our established methods 45.

### Explant model of avascular meniscus tear healing

Our well-established meniscus explant model was used to investigate the effect of 4-PPBP on the healing of avascular meniscus injuries 13-15. Briefly, the inner 1/3 avascular zone of the meniscus was harvested from healthy adult bovine knee joints (#8-240128, n=12 per group, Animal Technologies, Tyler, Texas). In the middle of the inner avascular zone, a full-thickness longitudinal or radial tear was created, followed by gluing the incised tissues using FibGen bioglue, with or without 10 µM 4-PPBP or 200 µM BD1047. After surgical suture repair of the tears, human syMSCs (1M/ml) were applied on the meniscus lesion, allowing them to migrate into the repair site. The meniscus explants were then cultured in fibrochondrogenic supplements 13-15 for 4 weeks. The harvested tissues were analyzed using H&E, Safranin-O/fast green (Saf-O/FG), and Picrosirius Red (PR) staining with polarized microscopy.

Mechanical properties of the healed menisci were measured using a pull-out test per our prior methods 15. Briefly, upon mounting with tensile jigs in an isotonic saline bath, a 0.02-N tare load was applied to the samples, and then the samples were elongated at 10%/min until failure. From the force vs. elongation curve, the ultimate strength and tensile modulus were obtained. The tensile modulus was calculated as the slope of stress (force/cross-sectional area) vs. strain (displacement/initial length), and the ultimate strength was defined as the maximum load divided by the cross-sectional area. All pull-out tests were performed using Electroforce^®^ BioDynamics^®^ system.

### Animal study

All animal procedures were approved by the Institutional Animal Care and Use Committee (IACUC) at Columbia University (protocol number# AC-AACB7704). A total of 84 male Sprague-Dawley rats (250 to 275g, Charles River, Boston) were used approximately 1 week before the procedure to allow adequate animal acclimation. Given the 3 - 4 times higher incidence rate of meniscus injuries in males than females, this study tested the efficacy of 4-PPBP in males. There were 6 groups of animals: normal control, sham control, nontreated meniscal defect, bioglue only, bioglue with 10 µM 4-PPBP, bioglue + 4-PPBP + 200 nM BD1047 (n=6~8 in each group per time point; 2 and 4 weeks).

For surgeries, animals were placed in an induction chamber with isoflurane (#1169-8777-2, Covetous, USA) administration at 4% for induction of anesthesia before placement on a surgical table. Sustained-release buprenorphine was administered before surgery. Continuous isoflurane administration at 1.5-2% was used for maintenance of anesthesia. The anesthetic state was assessed using a hind-limb toe pinch and respiratory observation. The state of anesthesia was continuously monitored (pinch reflex) throughout the procedure. The surgical site was shaved, prepped with povidone-iodine/ethanol, and draped in a sterile surgical manner as we described previously 46. Each rat was positioned in the supine position to expose the meniscus after hair removal and 3 surgical disinfections. The right knee was shaved and prepped with povidone-iodine/ethanol and draped in a sterile surgical manner. Rats were placed on a warm water-flowing heating pad to maintain body temperature. A 1.5 cm vertical incision was made and then the medial joint capsule was sectioned. Before the defects were created, all menisci were confirmed to have a sufficient size for creating the planned defects. A 2 mm complete meniscus injury was performed in the inner two-thirds of the anterior portion of the medial meniscus. 20 μl bioglue was injected between the injury sites. The skin was sutured continuously with absorbable sutures. 2 rats per cage were housed under pathogen-free conditions. They were fed standard laboratory rodent and water *ad libitum*. Animals were maintained at a constant temperature of 25°C and kept on a 12-h light/dark cycle. Finally, rats were euthanized by carbon dioxide inhalation at each time point.

### Evaluation of joint functions

Gait analysis was performed using a custom-built CatWalk system, with video-based functional assessment. Animals were allowed to walk at least 5 times through a one-meter-long treadmill, equipped with a video recording system 47. Average walking speed, stance length, stride length, and stance width were evaluated for gait analysis. The passive range of motion was also measured according to previous methods 48. Briefly, after clamping the femur onto an arthrometer, a fixed torque of 23.4 N⋅cm was applied. A picture was captured with a mounted camera to measure the ROM. The measurements were repeated 3 times for each knee sample.

### Histological staining

Rat knee joints were fixed, decalcified, embedded in paraffin, and coronally sectioned (5 µm). For each knee joint, 9 slides at 50 µm intervals were created which were stained with H&E for morphologic analysis. For histomorphometric measurement of meniscus and articular cartilage area, the SO-positive staining areas of four quadrants were traced, and the size of each selected area was calculated using ImageJ system. The severity of cartilage damage was assessed by analyzing all four quadrants of the joint, including medial femoral, medial tibial, lateral femoral, and lateral tibial cartilage, using the Osteoarthritis Research Society International (OARSI) score system by two blind observers. The final OARSI and articular cartilage area scores were the average of the four quadrants across the two observers. The meniscus repair score was assessed by using the Pauli score 49. Picrosirius red staining was applied to detect collagen fiber formation according to the manufacturer's manual (#24901, Polysciences, USA).

### Immunofluorescence (IF)

Immunofluorescence for σ1R and MMP-3 was measured in syMSCs and knee joint sections treated by 4-PPBP with or without σ1R antagonist, BD1047. Briefly, primary cells were fixed with 4% paraformaldehyde for 20 minutes and then rinsed with D-PBS (calcium and magnesium) 3 times. Permeabilize the cells with 0.3% Triton-X for 5 minutes at room temperature before 5% BSA blocking and antibody incubation. All samples were blocked with BSA (D9663, Sigma, USA) for 1 h to eliminate non-specific binding of the primary antibody. Subsequently, the samples were incubated with primary antibodies for σ1R (ab53852, Abcam) or MMP-3 (ab53015, Abcam) (ratio 1:200) overnight at 4°C, then fluorochrome-labeled secondary antibodies (Alexa Fluor 488, 115-025-003, ratio at 1:200) at 37°C for 1.5 h. Nuclei were stained with 4,6-diamidino-2-phenylindole (DAPI, ratio at 1:5000) for 15 min at room temperature. Images were captured using a fluorescence microscope (Nikon Eclipse, Tokyo, Japan).

### Single-cell RNA sequencing and CellChat analysis

At 2 weeks post-op, cells were isolated from whole knee joint tissues (n = 3 per group), including synovium, ligament, infrapatellar fat pad, cartilage, and medial meniscus. The harvested tissues were minced, followed by 5 mg/ml collagenase I and IV in serum-free DMEM for 2.5 h at 37°. After enzymatic digestion, an equal volume of 10% FBS in DMEM was added to neutralize the enzymes. Suspended cells were transferred to a 100 µm strainer, centrifuged at 1,600 rpm, and rinsed 3 three times using PBS with 10% FBS. All cells isolated from 3 animals were combined for scRNA-seq analysis. Library construction and scRNA-seq were performed on the 10XGenomics platform. The scRNA-seq analysis was conducted in R (version 4.4.2) with a rigorously developed pipeline integrating multiple analytical tools. Raw count matrices were imported from HDF5 files, from which the Seurat objects were generated separately for each sample using Seurat (version 5.2.1). Quality control steps entailed filtering out cells with fewer than 200 or more than 6,000 detected features and with greater than 20% mitochondrial gene expression. Gene expression data were then globally normalized, and the top 2,000 variable genes were identified using a variance-stabilizing transformation. Scaled data were subjected to principal component analysis (PCA). The number of principal components used in downstream analyses was determined via the elbow plot by identifying the inflection point at which additional principal components contributed only marginally to the variance. Doublet detection was performed using DoubletFinder (version 2.0.4) with an estimated doublet rate of approximately 7.5%, and only cells classified as singlets were retained. Following normalization and filtering, control and OP datasets were merged and batch-corrected using Harmony (version 1.2.3) with the original sample identifier as the grouping variable. Clustering was conducted based on the optimal number of PCA components (as determined by the elbow plot) and subsequently visualized using both UMAP, produced via ggplot2 (version 3.5.1) and patchwork (version 1.3.0). Cell type annotation was initially performed via differential expression analysis using Seurat's FindAllMarkers function (with a log₂ fold-change cutoff of 0.25 and a minimum percentage threshold of 25% for gene expression).

To elucidate intercellular communication, a dedicated Seurat object was constructed for CellChat analysis 50 using CellChat (version 1.6.1) and associated packages (circlize version 0.4.16, grid version 4.4.2, and Matrix version 1.7.3), with a rat-specific CellChat database 51. Custom visualization functions were developed to generate two types of plots: a circular network plot and chord diagrams. The circular network plot displays the global cell-cell communication landscape, where vertex sizes indicate cell population sizes and edge weights reflect the strength of communication probabilities. The chord diagram is designed to illustrate specific signaling interactions, highlighting the selected signaling pathways based on interaction scores or selected pathway of interest. In these chord diagrams, chord thickness denotes the communication strength between cell types, offering insights into the key ligand-receptor mediated interactions. Quantitative comparisons of cell-cell communication signals were performed between the control and 4-PPBP treatment groups, using comparative CellChat analysis. Briefly, cellchat objects created from control and treatment samples were merged into a new cellchat object. The merged cellchat object was analyzed for cell-cell communications, and significantly upregulated or downregulated cell-cell signaling pathways were identified for each cell-cell pair, followed by visualization with bubble plots. Differential expression analyses between the control and treatment groups within each annotated cell type were further carried out, and significant changes were visualized using volcano plots, heatmaps, and dot plots.

### Statistical analysis

Statistical analyses were performed using GraphPad Prism (version 10.2.3) for all quantitative data. A one-way Analysis of Variance (ANOVA) with post-hoc Bonferroni tests was applied with a significance level set at P < 0.05. All data are presented as means ± SD, unless stated otherwise. To ensure scientific rigor and unbiased data analysis, all qualitative and quantitative assessments were conducted in a blinded manner.

## Discussion

Our study demonstrated the notable potential of a novel multi-functional small molecule, 4-PPBP, to enhance the integrative healing of avascular meniscus tears by concurrently regulating inflammation and stimulating endogenous stem/progenitor cells. Our collective data supports the functions of 4-PPBP in stimulating migration, proliferation, and differentiation of syMSCs and in reducing inflammation. As the inflammatory cascade caused by meniscus injuries plays a critical role in the delayed healing and initiation of meniscus degeneration, anti-inflammatory modulation is essential for the functional restoration of meniscus injuries 52. To provide multiple functions of inflammatory modulation and matrix synthesis, previous approaches have incorporated multiple bioactive ingredients (e.g., bioactive materials, cells, and growth factors) in meniscus bioactive glues 13-15, 23, 53. However, such a complex design of multifunctional biologics-releasing hydrogels poses inevitable translational barriers, including regulatory restrictions and developmental costs 17. Thus, our single molecule, which exhibits promising anti-inflammatory effects and activates syMSCs, may present notable translational potential. In addition, 4-PPBP can be delivered into the synovial joint using a simple, injectable fibrin-based hydrogel crosslinked with a plant-derived, safe crosslinker, further advocating the translational readiness of our approach.

We have confirmed that 4-PPBP functions through σ1R, leading to meniscus healing and modulating inflammation. σ1R is a unique chaperone protein primarily located in the endoplasmic reticulum (ER), particularly in the mitochondria-associated membrane (MAM), the interface between the ER and mitochondria. Likely, σ1R plays an essential role in maintaining ER-mitochondrial communication, especially during cellular stress and in regulating calcium signaling. As σ1R is present in many types of joint cells, including syMSCs, fibrochondrocytes, chondrocytes, fibroblasts, and macrophages, the multiple functions of σ1R may be exhibited through multiple types of cells. Likely, our scRNA-seq and CellChat analysis showed that numerous genes were significantly up- or down-regulated in each type of joint cells by 4-PPBP delivery. Consistently, previous studies reported the distinct functions of σ1R in different types of cells under various circumstances 31, 32. Nonetheless, it is also plausible that σ1R exerts multiple functions through each type of cell, supported by our *in vitro* data demonstrating anti-inflammatory effect and differentiation induction in syMSCs. Similarly, previous studies elucidated various roles of σ1R in conjunction with various signaling pathways 54, 55. Yet, we cannot rule out the possibility that multiple functions of 4-PPBP presented *in vivo* may be presented through indirect interactions among various types of cells and 4-PPBP-stimulated cells. Although we discovered that multiple types of cells are responding to 4-PPBP delivery, either directly or indirectly, we do not have a clear understanding of the cell type-specific mechanism of 4-PPBP's function through σ1R, representing a limitation of this study.

Our scRNA-seq analysis with CellChat revealed interesting cell-cell communication in joint tissues treated with 4-PPBP. While joint connective tissue cells, such as fibrochondrocytes, fibroblasts, and chondrocytes, are primarily involved with anabolic signals, adipocytes robustly increased cell-cell communication signals to macrophages associated with anti-inflammatory modulation. 4-PPBP delivery significantly increased ApoE signal from adipocytes to macrophages. Given the reported role of ApoE in promoting M2 polarization, the elevated ApoE signals observed with 4-PPBP are likely to modulate inflammation by altering macrophage polarization. The CellChat-identified roles of adipocytes in modulating macrophage polarization are likely consistent with the increasing body of research on the roles of the infrapatellar fat pad (IFP) in synovial joint inflammation and initiation/progression of PTOA 56, 57. Interestingly, our CellChat analysis revealed another cell-cell communication leading to anti-inflammatory polarization of macrophages. With 4-PPBP delivery, fibrochondrocytes significantly increased Tn-C & Tn-N to Sdc4 signal axis to macrophages. Given the role of Tn-C and Tn-N in promoting M2 polarization and reducing inflammation 37, this observation suggests the novel anti-inflammatory function of fibrochondrocytes through their interactions with macrophages. The anti-inflammatory function of meniscus fibrochondrocytes has rarely been investigated 58, 59.

Limitations of this study include the lack of mechanical evaluation of *in vivo* healed menisci. Due to the small size of the harvested rat menisci, it was technically infeasible to perform conventional mechanical tests for the functional restoration of meniscus tissues. Although micro-scale measurements, such as nanoindentation, can measure indentation moduli and surface congruency, the compressive and tensile moduli of the healing meniscus can hardly be measured. Thus, we performed a gait analysis to evaluate the functional outcome of meniscus healing. Despite being indirect, our gait measurement indicated the promising functional restoration of the joint with 4-PPBP application, although such gait function and ROM are potentially influenced by other factors such as pain, inflammation, and joint stiffness. A relatively short-term in vivo follow-up is another limitation of this study. Although 4-week was sufficient to observe the initial healing of meniscus tears, it may not represent long-term stability of the healing. Our future study will include a long-term follow-up study. Another limitation of this study is the limited methodology to validate the roles of the cell-cell communication signal pathways identified by scRNA-seq and CellChat analysis with *in vivo* samples. *In vitro* co-culture experiments with selected signaling inhibitors may be explored to validate the cell-cell communication signals 60, 61. However, cell-cell communication signals are likely involved in the systemic network of multi-tissue interactions, rather than communications between selected pairs of cell types 62, 63. Thus, the currently available technology is limited in its ability to recapitulate the complex, *in vivo* multi-tissue crosstalk that regulates meniscus healing and inflammatory modulation. Emerging new approach methodologies (NAMs), such as joint-on-a-chip (JoC), can be applied to validate the cell-cell signaling pathways identified in this study once the technology reaches acceptable quality.

To conclude, the multi-functional small molecule, 4-PPBP, delivered via FibGen bioglue, demonstrates its potential as a novel regenerative therapeutic for meniscus injury. 4-PPBP-loaded FibGen bioglue effectively enhances meniscus healing and delays degeneration by modulating multi-tissue crosstalk. These findings may represent a breakthrough in meniscus treatment, helping patients restore mobility, improve function, and lead healthier, more fulfilling lives.

## Supplementary Material

Supplementary figures.

## Data Availability

The scRNA-seq data generated and analyzed in this study are available in GEO repository (GSE317855).
