# Supplementary material for: Multi-functional Small Molecule for Regenerative Healing of Avascular Meniscus Tears: Modulation of Inflammation, Differentiation, and Multi-Tissue Crosstalk
Source: Theranostics. 2026 Apr 8;16(11):5911–25. doi: 10.7150/thno.132326 (PMC13142125; doi:10.7150/thno.132326)
Supplement: Supplementary file 1 — Supplementary figures. [file thnov16p5911s1.pdf]

Meng et al., 2026, Supplementary Data

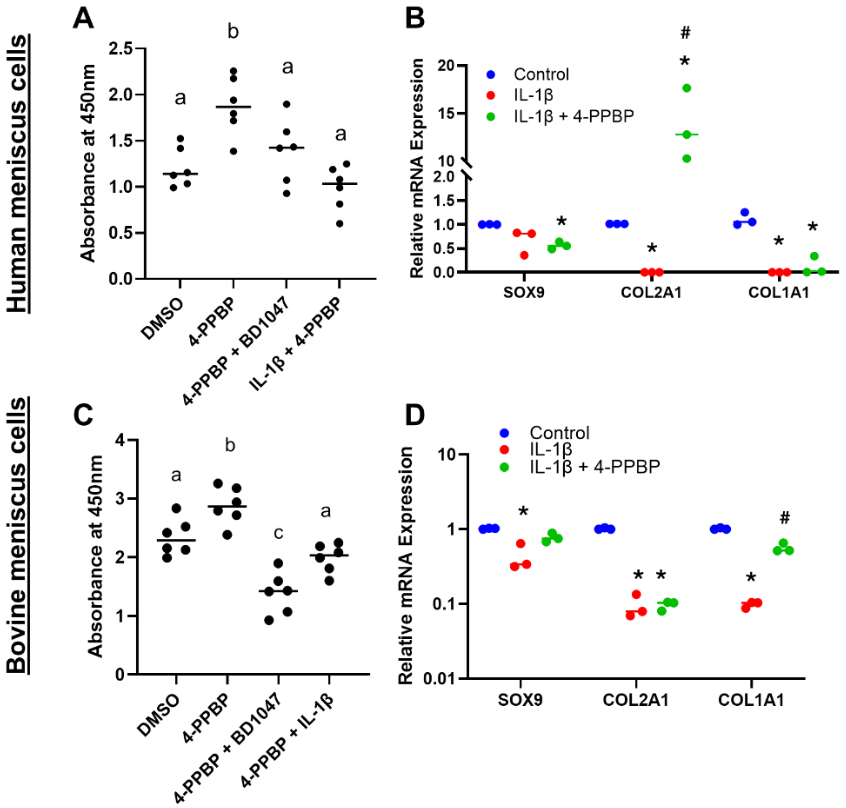

Supplementary Figure 1. Anti-inflammatory function of 4-PPBP in human and bovine meniscal cells: CCK-8 assay (**A, C**) (different letters indicate significant difference; p<0.001) and qRT-PCR (**B, D**) (*: p<0.01 compared to control; #: p<0.01 compared to all the other groups).

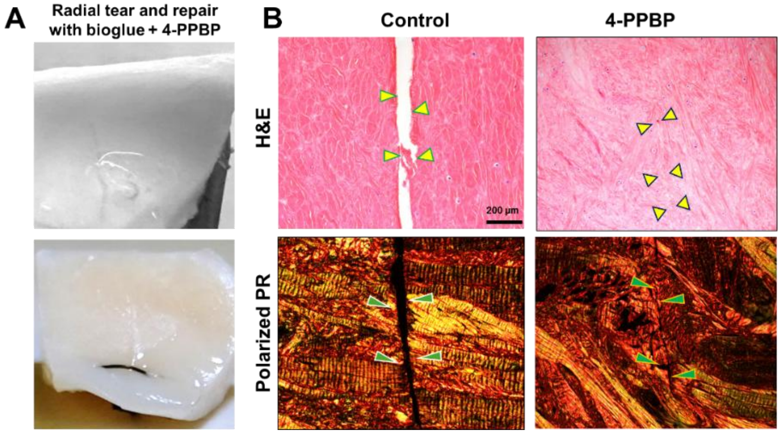

Supplementary Figure 2. Healing of radial tears in meniscus explants by 4-PPBP. Full-thickness radial tears were created at the inner avascular zone of bovine meniscus explants, followed by all-inside suture repair with 4-PPBP/FibGen treatment (**A**). After 4 weeks, 4-PPBP treatment resulted in integrated healing of fibrocartilaginous tissue as compared to the remaining gap in the control with FibGen alone (**B**).

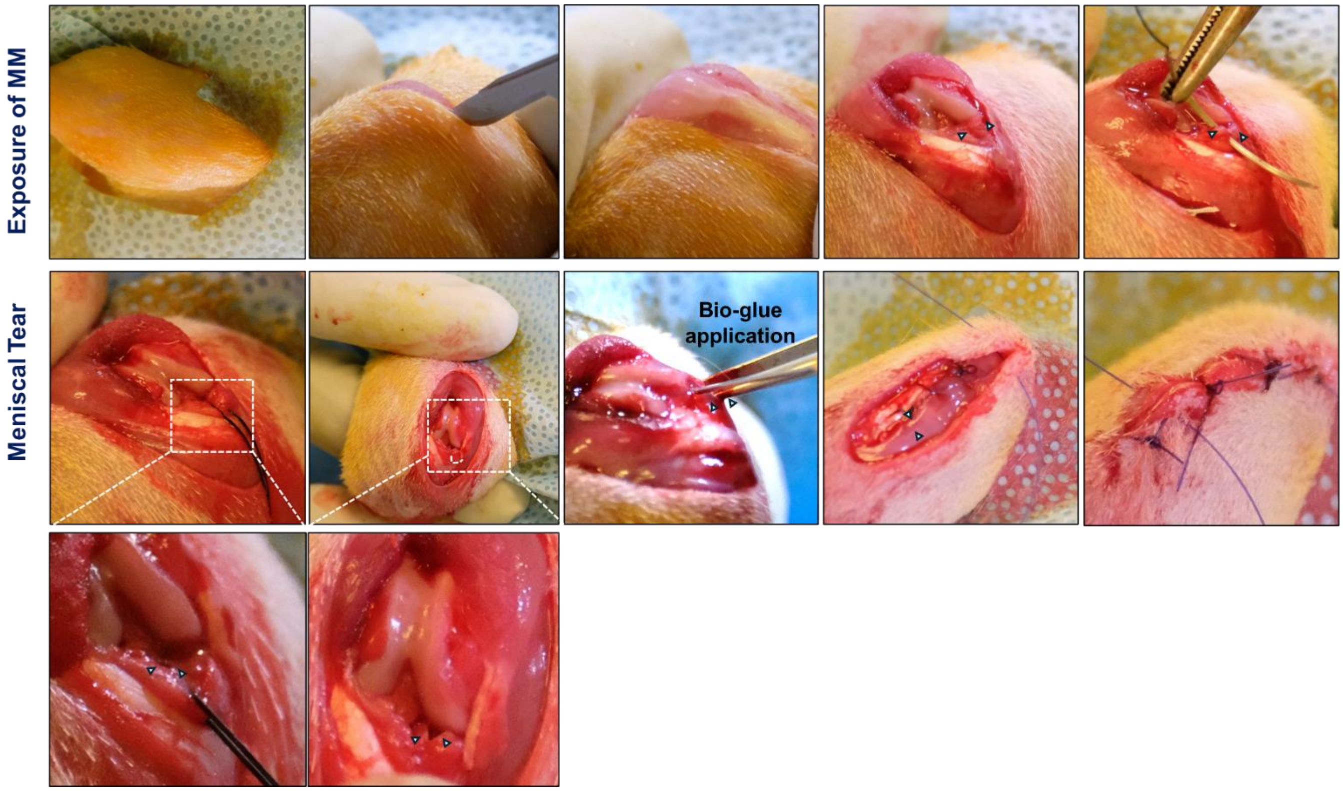

Supplementary Figure 3. Surgical procedure to create longitudinal tears in anterior portion of rat meniscus, followed by application of bioglue.

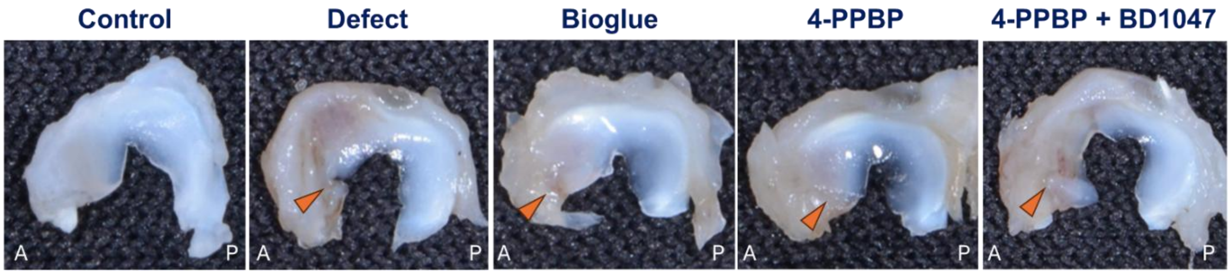

Supplementary Figure 4. Macroscopic evaluation of rat menisci harvested 2 weeks post-op (A: anterior, B: Posterior; arrow: defect).

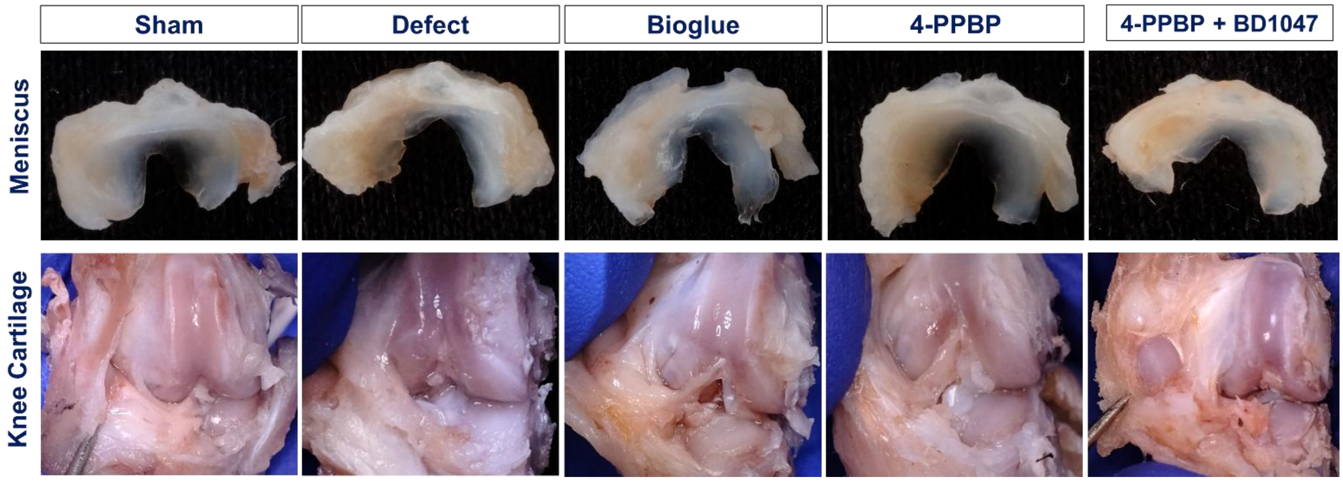

Supplementary Figure 5. Macroscopic evaluation of meniscus and cartilages at 4 weeks post-op.

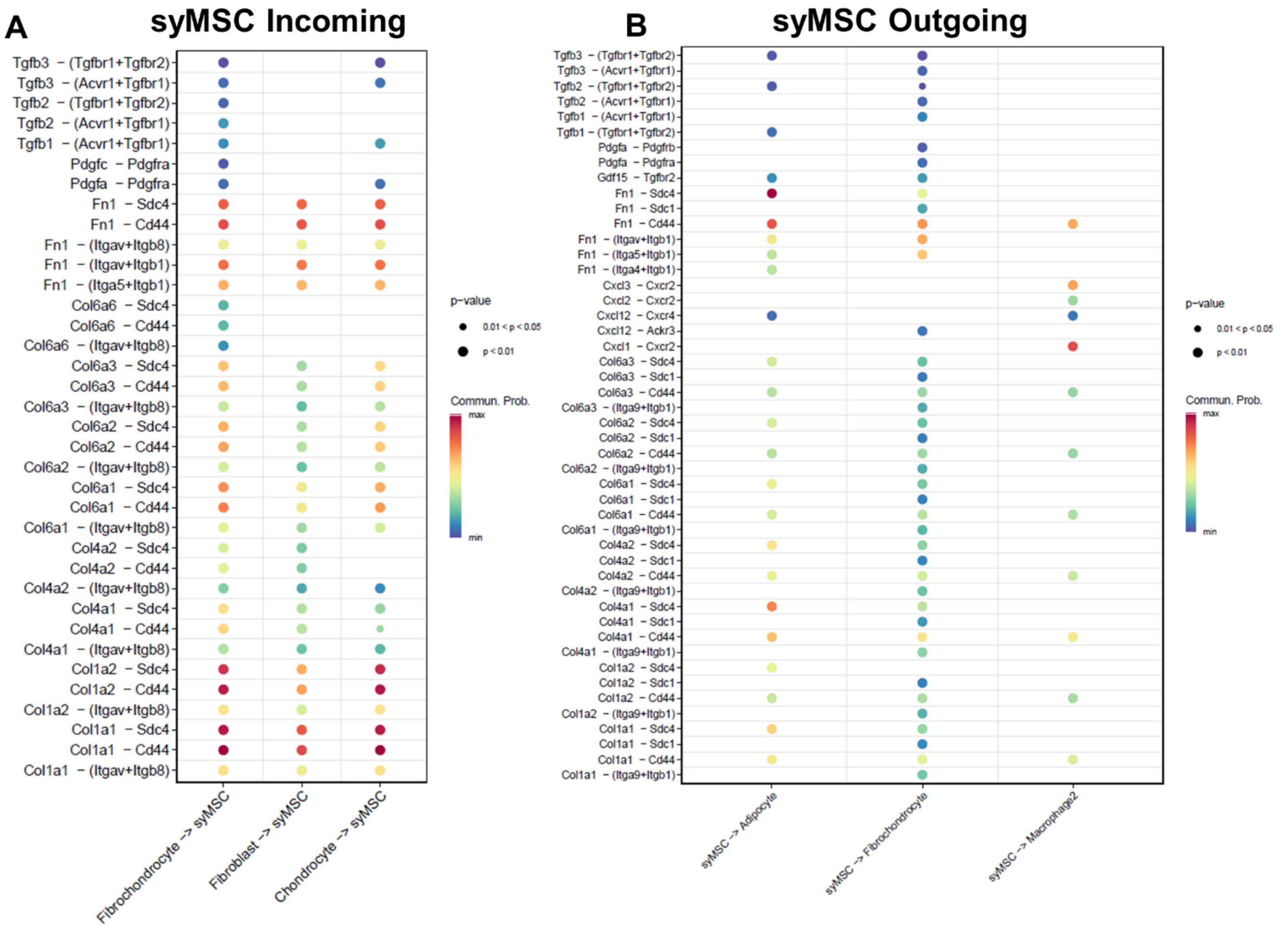

Supplementary Figure 6. Bubble plots showing robust cell-cell communication signals involved with syMSCs in the 4-PPBP treatment group.

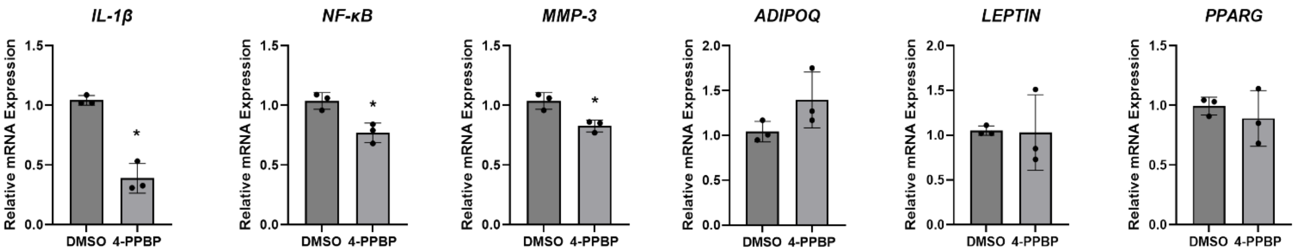

Supplementary Figure 7. Effect of 4-PPBP on adipocytes *in vitro*. (n = 3 per group; *:p<0.01 compared to DMSO control).

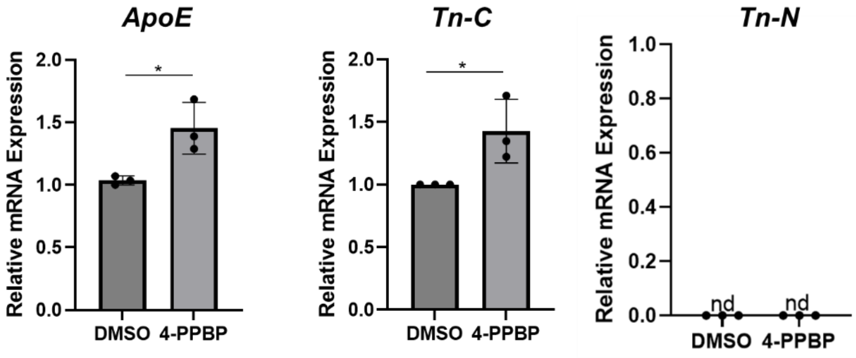

Supplementary Figure 8. ApoE, Tn-C, and Tn-N expressions in fibrochondrocytes with 4-PPBP treatment (n = 3 per group; *:p<0.01).
